# Design and Dynamic In Vivo Validation of a Multi-Channel Stretchable Liquid Metal Coil Array

**DOI:** 10.3390/ma17133325

**Published:** 2024-07-05

**Authors:** Elizaveta Motovilova, Terry Ching, Jana Vincent, Ek Tsoon Tan, Victor Taracila, Fraser Robb, Michinao Hashimoto, Darryl B. Sneag, Simone Angela Winkler

**Affiliations:** 1 Department of Radiology, Weill Cornell Medicine, New York, NY 10065, USA; 2 Department of Radiology and Imaging, Hospital for Special Surgery, New York, NY 10021, USA; 3 Pillar of Engineering Product Development, Singapore University of Technology and Design, Singapore 487372, Singapore; 4 Digital Manufacturing and Design (DManD) Centre, Singapore University of Technology and Design, Singapore 487372, Singapore; 5 Department of Biomedical Engineering, National University of Singapore, Singapore 117583, Singapore; 6 GE Healthcare, Aurora, OH 44202, USA

**Keywords:** RF coil, MRI, liquid metal, stretchable

## Abstract

Recent developments in the field of radiofrequency (RF) coils for magnetic resonance imaging (MRI) offer flexible and patient-friendly solutions. Previously, we demonstrated a proof-of-concept single-element stretchable coil design based on liquid metal and a self-tuning smart geometry. In this work, we numerically analyze and experimentally study a multi-channel stretchable coil array and demonstrate its application in dynamic knee imaging. We also compare our flexible coil array to a commonly used commercial rigid coil array. Our numerical analysis shows that the proposed coil array maintains its resonance frequency (<1% variation) and sensitivity (<6%) at various stretching configurations from 0% to 30%. We experimentally demonstrate that the signal-to-noise ratio (SNR) of the acquired MRI images is improved by up to four times with the stretchable coil array due to its conformal and therefore tight-fitting nature. This stretchable array allows for dynamic knee imaging at different flexion angles, infeasible with traditional, rigid coil arrays. These findings are significant as they address the limitations of current rigid coil technology, offering a solution that enhances patient comfort and image quality, particularly in applications requiring dynamic imaging.

## 1. Introduction

Magnetic resonance imaging (MRI) has evolved as a powerful diagnostic tool, offering non-invasive insights into the internal structures of the human body with exceptional spatial resolution [1]. The pursuit of improving MRI technology has led to innovations [2,3,4] in radiofrequency (RF) receive coils, a critical component influencing image quality and signal-to-noise ratio (SNR) [5].

Recent advancements in RF coil design and development have been significantly influenced by the goal of enhancing patient comfort, leading to the creation of numerous flexible and stretchable solutions [4]. The flexibility of these coils varies based on the design methodology and the materials used. For instance, one approach involves printing a thin and flexible coil on both sides of a Kapton substrate to incorporate the necessary inline capacitors for coil resonance [6]. The screen-printing fabrication technique developed by Corea et al. [7] eliminates the need for bulky copper wires, porcelain capacitors, and thick substrates, resulting in greater flexibility and closer coil placement, particularly advantageous for pediatric imaging [8]. Jia et al. used a flexible ribbon cable to form a resonant coil [9]. Several semi-rigid coil arrays have been designed to wrap around 2D curved surfaces [10,11,12] or to be mechanically adjusted for better fit [13,14,15,16,17], accommodating different patient sizes. High-impedance coil (HIC) technology [18,19], coaxial cable coils [20,21,22], and multi-turn cable coils [23] offer enhanced inter-element isolation, flexibility, and even modular adaptive configurations [24], are proving useful for dynamic imaging applications. Additionally, GE Healthcare’s commercially available adaptive image receive (AIR) coil technology [25,26] is ultra-lightweight, free from lumped components on the coil conductor, and easily conforms to various patient anatomies like a blanket, further advancing the flexibility and usability of RF coils.

Among these innovations, non-traditional conductive materials such as copper braids [27], silver-coated threads [28], liquid metal [29,30,31,32,33], and conductive elastomers [34] have been proposed as alternative materials for RF coil fabrication due to their flexibility and adaptability to anatomical variations and potential to accommodate dynamic imaging scenarios. For example, braided copper coils [27] were used for knee imaging under varying flexion angles up to 60° while maintaining uniform coverage of the entire joint. However, constructing stretchable braided copper coils is challenging because they must be integrated with an elastic substrate that accurately returns the rigid conductor to its original shape after deformation. Another method uses conductive silver thread sewn onto a stretchable textile [28], which simplifies manufacturing but requires large amounts of thin yarn to achieve adequate electrical performance, posing mechanical challenges. Gallium (Ga) liquid metal and its alloys, such as eutectic GaIn (eGaIn) (75.5% Ga, 24.5% In) and Galinstan GaInSn (68.5% Ga, 21.5% In, 10% Sn, by weight), have recently gained a lot of attention as they are intrinsically stretchable, have high electrical conductivity (3.4 × 10^6^ S/m), low viscosity (2.4 mPa·s), negligible vapor pressure [35], and are biocompatible [36]. Several research prototypes have been made where Ga-based liquid metal was used as an ink in stencil printing on stretchable neoprene fabric [30,31,32]. However, limited thickness of stencil-printed liquid metal coil results in lower electrical performance, leaving liquid metal exposed and prone to smearing. One way to contain liquid metal is to use it as a filler inside stretchable silicone tubes [29,33]. In [34], silver microparticles were mixed with an elastomer to form stretchable conductive wires. Although these alternative materials tend to have higher losses compared to traditional copper conductors, research has demonstrated that they can achieve similar signal-to-noise ratios (SNRs) and offer additional benefits like reduced weight and radiological transparency [37].

Despite their promising attributes, the effectiveness of these stretchable coils can be compromised due to changes in the resonance frequency caused by a change in coil inductance when stretching conductors. This phenomenon poses a challenge to maintaining the SNR advantages associated with close-fitting receive coils. To address this issue, various solutions have been proposed, including field programmable gate array (FPGA)-based tuning/matching circuits [32], low-variability preamplifiers [38], and π-matching networks [14,33]. In response to this challenge, we previously introduced a self-tuning interdigital capacitor geometry to offset inductance change with commensurate capacitance change [39,40]. We demonstrated the performance of a single coil element prototype in silico and in vivo, confirming its ability to mitigate the inherent frequency shift associated with stretching up to 30%. To the best of our knowledge, this is the only research coil prototype that addresses the frequency detuning issue associated with stretchable MRI coils from the coil design perspective as opposed to external circuit design.

Building upon these advancements, the current research aims to extend the application of self-tuning techniques to a multi-channel stretchable coil array. The transition from a single element to a multi-element array (i.e., multi-channel) is crucial for practical implementation in imaging systems as most of MRI coils utilize multi-element configurations to cover a larger area of interest and to enable parallel imaging [41]. The ability to stretch the coil and adapt it to varying anatomies while maintaining optimal resonance frequency bears the promise of dynamic MRI imaging, offering unprecedented flexibility in diagnostic procedures. Additionally, soft and form-fitting coils provide improved patient comfort and could ultimately result in improved patient compliance.

This paper presents a comprehensive investigation into the feasibility and performance of a multi-channel, stretchable, self-tuning, liquid metal-based RF receive coil array. Our objectives include optimizing the array design through numerical simulations, employing optimized, state-of-the-art fabrication techniques based on previous work [42], and conducting in vitro and in vivo experiments to assess the array’s performance in dynamic imaging scenarios. The study aims to bridge the gap between theoretical simulations and real-world applications, showcasing the potential clinical relevance of this innovative coil design. We hypothesize that our multi-channel stretchable RF coil array based on liquid metal and a self-tuning mechanism will maintain optimal resonance frequency and SNR under varying stretching configurations, thereby offering superior image quality and greater adaptability for dynamic MRI imaging compared to traditional rigid coil arrays.

The paper is organized as follows: Section 2 details the methods employed, including numerical simulations, fabrication processes, and imaging experiments. Section 3 presents the results obtained from simulations and in vitro and in vivo experiments. Section 4 discusses the implications of the findings and potential clinical applications, self-tuning array advantages, as well as limitations. Section 5 concludes the paper and outlines avenues for future research, emphasizing the ongoing pursuit of optimizing and expanding the capabilities of flexible RF receive coil arrays for MRI imaging that is oriented towards optimized performance and patient comfort. Through our comprehensive investigation, we seek to contribute to the evolving landscape of RF coil technology, paving the way for advancements that can significantly impact the field of medical imaging and patient treatment.

## 2. Materials and Methods

### 2.1. Simulations

**Figure 1:**
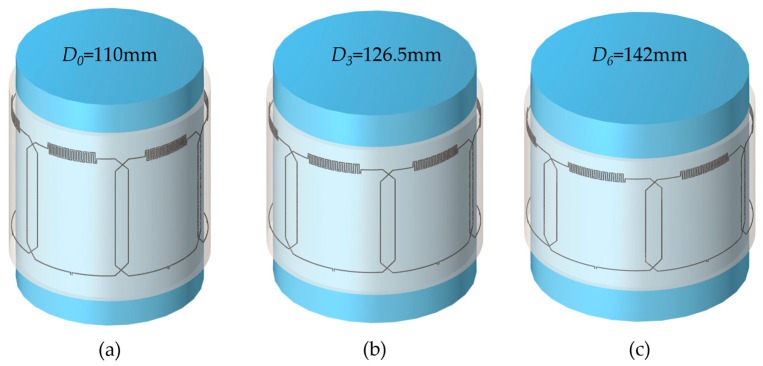
A 3D model of the 6-channel coil array wrapped around cylindrical phantoms of different diameters, (**a**) *D*_0_ = 110 mm (**b**) *D*_3_ = 126.5 mm, and (**c**) *D*_6_ = 143 mm, representing radial stretching of 0%, 15%, and 30%, respectively.

Numerical simulations were conducted using COMSOL Multiphysics 6.2 (Burlington, MA, USA) to optimize the design and performance of the multi-channel stretchable array. The Electromagnetic Waves, Frequency Domain COMSOL module was used. The coil array comprised six elements, each featuring a 7 cm × 8 cm rectangular loop with an integrated interdigital self-tuning capacitor [39]. The conducting traces were implemented as perfect electric conductors (PECs) of 0.5 mm × 0.5 mm cross-section, embedded in a dielectric polymer substrate (dielectric constant, ε = 2.7). To ensure decoupling between neighboring elements, a critical overlap distance of 12 mm was implemented based on previous *S*_21_ simulations of a dual-channel coil array [43]. The coil array was wrapped around a homogeneous cylindrical phantom (dielectric constant, ε = 78, electrical conductivity, σ = 0.46 S/m). The coil array was simulated in various stretching configurations using cylindrical phantoms with a height of 150 mm and different diameters ranging from *D*_0_ = 110 mm to *D*_6_ = 143 mm, representing varied degrees of stretch from 0% to 30%, with a step size of 5%. The dimensions of the coil array were altered axially with radial stretching using the numerical approximation stretching formula previously proposed in [42], where all coil geometry elements were parameterized with dimensionless variables $\lambda_{x},\lambda_{y},\lambda_{z},$ where the subscripts correspond to the three Cartesian directions *x*, *y*, and *z*, respectively. First, the coil element was modeled in SOLIDWORKS 2024 (Dassault Systèmes, Vélizy-Villacoublay, France) on a 2D *xy*-surface and uniaxially stretched along the *x*-direction by changing the variable $\lambda_{x}$ from 1 to 1.3, representing elongations from 0% to 30%. Assuming the materials are elastically homogenous and virtually incompressible, the following constraint is imposed $\lambda_{x}\cdot \lambda_{y}\cdot \lambda_{z}=1$, from where it follows that *y* and *z* dimensions will change (shrink) according to $\lambda_{y}=\lambda_{z}=1/\sqrt{\lambda_{x}}$. Next, the stretched coil element is wrapped around an auxiliary cylinder with the correspondingly increased diameter using the wrap feature. Finally, the now curved coil element is exported into COMSOL for further numerical electromagnetic simulations. Figure 1 shows the 3D model of the 6-channel array wrapped around three representative phantoms of diameter (a) *D*_0_ = 110 mm, (b) *D*_3_ = 126.5 mm, and (c) *D*_6_ = 143 mm, corresponding to coil array stretches of 0%, 15%, and 30%, respectively. More detailed simulation instructions are available in the Supplementary Materials Section S1.

### 2.2. Array Fabrication

**Figure 2:**
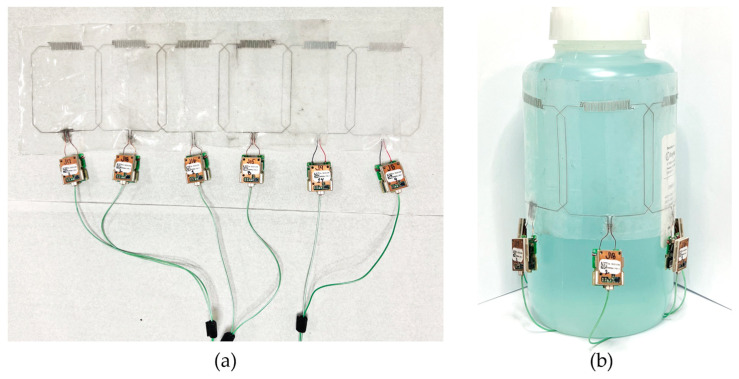
(**a**) Fabricated stretchable coil array. (**b**) Array wrapped around a standard phantom.

The fabrication process involved the use of a previously described direct ink writing (DIW) technique [42]. DragonSkin^TM^ 30 silicone (Smooth-On, Macungie, PA, USA) was spin-coated on a glass panel to create a thin 0.3 mm substrate. Microchannel walls were printed using a DIW printer (SHOTmini200ΩX, Musashi, Tokyo, Japan). A fast-curing silicone sealant (SpeedSeal, Selleys, Padstow, Australia) served as the liquid ink for the microchannel walls. A second layer of DragonSkin^TM^ silicone was pressed on top of the printed structure to seal the microchannels. eGaIn (Ga 75.5%/In 24.5%, Sigma-Aldrich, Merck, Rahway, NJ, USA) liquid metal was injected into the microchannels to create the conducting traces of the coil element. Copper wires were inserted at the terminals and connected to a printed circuit board containing tuning, matching, detuning, and preamplifier circuitry (GE Healthcare, Aurora, OH, USA). The individual coil elements were arranged into a linear, 1 × 6 array and attached to each other using fast-curing silicone adhesive (Sil-Poxy^TM^ by Smooth-On, Macungie, PA, USA). Figure 2a shows a photograph of the fabricated 6-channel array positioned on a flat surface with the total measured dimensions of the array of 38 cm × 8 cm. To form a cylindrical array, the first and last elements were also connected using Sil-Poxy^TM^ adhesive. Figure 2b shows a photograph of the cylindrical array wrapped around a standard homogenous phantom (L = 150 mm, D = 125 mm).

### 2.3. In Vitro Imaging

Imaging experiments were performed on a 3T MRI system (MR750, GE Healthcare, Waukesha, WI, USA) to evaluate the performance of the stretchable coil array in vitro. There was a fast spin echo (FSE) sequence with time to repeat (TR) = 3000 ms, time to echo (TE) = 17.4 ms, echo train length (ETL) = 16, field of view (FOV) = 24 cm, number of averages (NEX) = 1, bandwidth (BW) = 10.42 kHz, and slice thickness = 3 mm. The coil array was wrapped circumferentially around a standard, homogeneous, cylindrical phantom (L = 150 mm, D = 125 mm). The experiments investigated full array as well as individual elements performance in their native (unstretched) state.

### 2.4. In Vivo Imaging

For the in vivo experiments, informed consent was obtained from healthy volunteers under a locally approved IRB protocol. The study included two participants: one male and one female. The male participant was 25 years old, and the female participant was 29 years old. Imaging was performed on the same 3T MRI system where the proposed 6-channel stretchable coil array was compared to a standard 8-channel knee coil array (GE Healthcare). To acquire images, axial FSE sequence was used with TR = 537 ms, TE = 9 ms, NEX = 1, ETL = 16, slice thickness = 3 mm, FOV = 160 × 160 mm^2^, and an in-plane resolution of 0.3 × 0.3 mm^2^. Additionally, sagittal FSE images were obtained with TR = 548 ms, TE = 8.8 ms, NEX = 1, ETL = 3, slice thickness = 3 mm, FOV = 160 × 160 mm^2^, and an in-plane resolution of 0.3 × 0.3 mm^2^ to demonstrate adaptability of the coil in different knee positions, specifically at full extension and approximately 30 degrees’ flexion.

## 3. Results

### 3.1. Simulations

**Figure 3:**
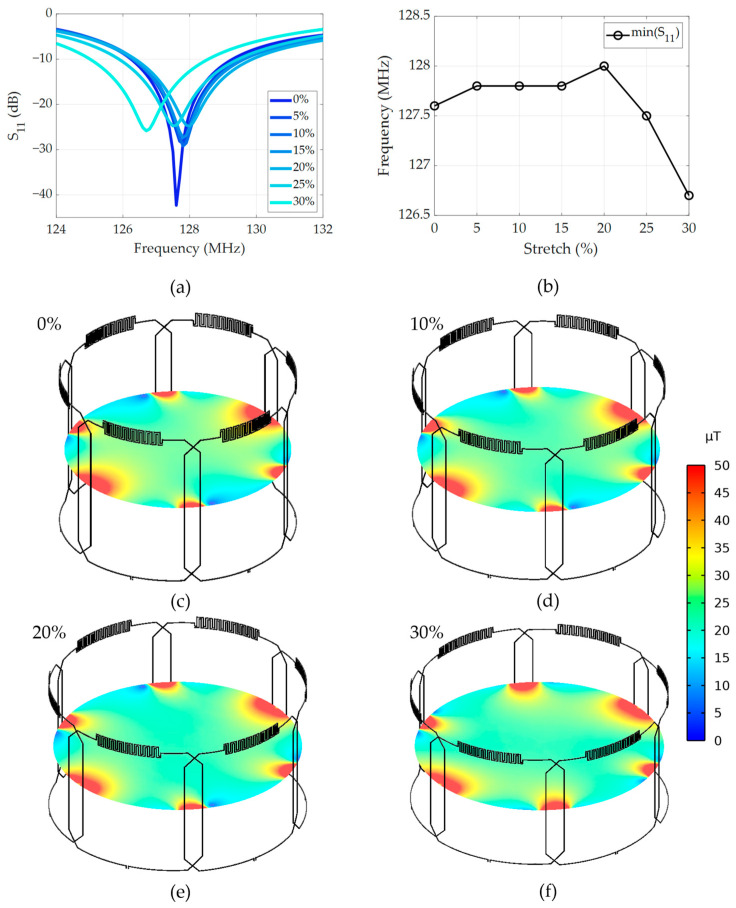
Simulated (**a**) S_11_ parameter, (**b**) resonance frequency, and (**c**–**f**) sensitivity changes with changing stretching/loading configurations.

Numerical simulations revealed robust performance of the multi-channel coil array. Figure 1a–c illustrate the 3D simulation model of the array in three representative stretching configurations with small, medium, and large cylindrical phantoms. Figure 3a shows the simulated S_11_ parameter changes for incremental stretching from 0% to 30% with a step size of 5%. The S_11_ parameter remains relatively stable around the resonance frequency of 127.8 MHz for stretching levels up to 25% and shifts the most at 30%, demonstrating relative frequency stability when stretched. Figure 3b presents the resonance frequency variation (measured as the S_11_ minimum) with stretching, indicating a negligible impact on the resonance frequency of less than 1 MHz from the center frequency of 127.8 MHz, aligning closely with previously published single-element stretching simulations [39]. Quantitatively, the average resonance frequency is calculated to be 127.6 MHz ± 0.43 MHz, which translates to less than 1% variation. Figure 3c–f show the combined sensitivity maps from all coil elements at four representative stretching and loading configurations (0%, 10%, 20%, and 30% stretch), which demonstrate a high degree of similarity of sensitivity. Specifically, the sensitivity (quantified as the absolute value of B_1_^-^ magnetic field strength) within the central axial slice of the phantom remains relatively stable, with an average magnetic field strength of 42.8 µT and a deviation of ±2.4 µT (±5.6%) when stretched.

### 3.2. In Vitro Imaging

**Figure 4:**
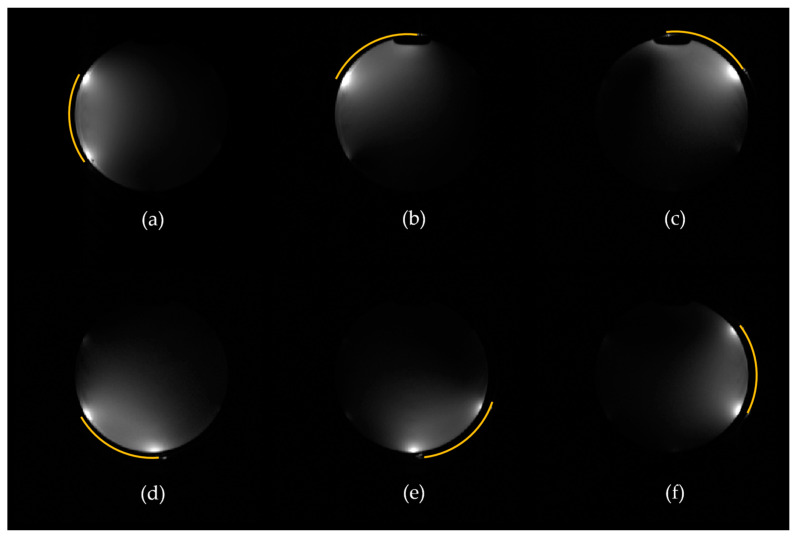
(**a**–**f**) Measured individual sensitivity profiles of stretchable coil elements 1 through 6. Yellow lines indicate the position of the corresponding coil elements.

**Figure 5:**
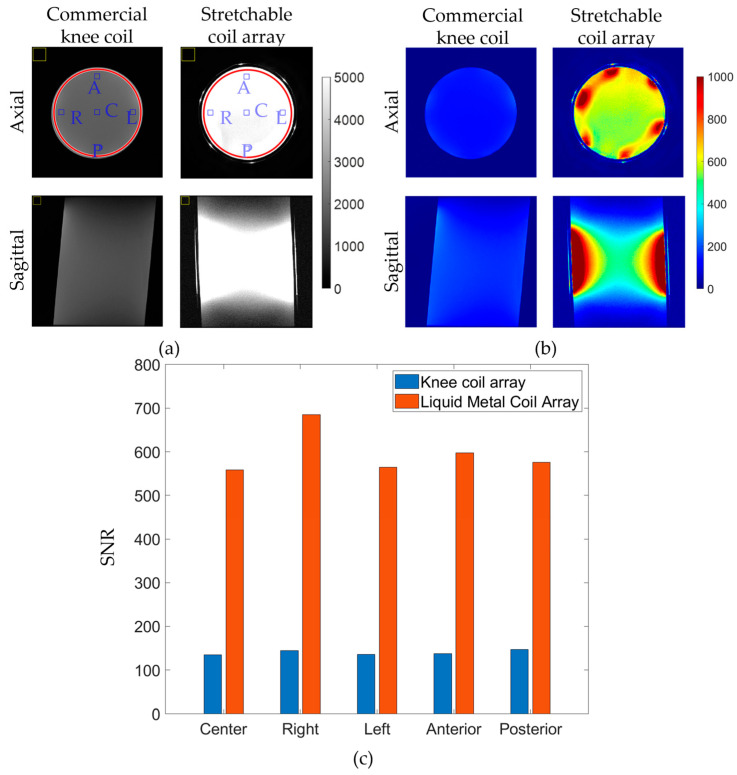
(**a**) Signal and (**b**) SNR measured using commercial knee coil and proposed stretchable coil arrays. (**c**) SNR comparison of the two coil arrays at the marked ROIs. Blue squares indicate smaller ROIs at central (C), right (R), left (L), anterior (A), and posterior (P) locations, and yellow squares indicate the positions where background noise was evaluated.

Figure 4a–f show the acquired individual signal (sensitivity) maps of all coil elements during in vitro experiments, demonstrating consistent performance across individual elements and agreement with simulations (Figure S2). Figure 5 provides a comparison of the stretchable six-channel liquid metal (LM) coil array and a commercial eight-channel rigid coil array in terms of measured signal and SNR. Figure 5 shows (a) signal and (b) SNR images for central axial and sagittal slices within a standard homogeneous cylindrical phantom. The noticeable signal improvement of the LM coil compared to the rigid coil is attributed to the tightly fitting design of the stretchable array, allowing for more signal to be captured due to its closer proximity. On the axial slices of Figure 5a, red circular lines indicate the region of interest (ROI) covering the entire cylinder, blue squares indicate smaller ROIs at central (C), right (R), left (L), anterior (A), and posterior (P) locations, and yellow squares indicate the positions where background noise was evaluated. The difference between the rigid coil and stretchable coil in terms of SNR within the entire cylinder (red lines) is calculated to be from 143 ± 9 (rigid coil) to 623 ± 108 (stretchable coil), indicating an average improvement of 4.4 times (or 440%). The increased deviation of the SNR of the stretchable coil is attributed to its close-fitting nature, as the SNR intensity at the periphery of the load is the highest due to the coil proximity. Figure 5c shows statistical differences in coil performance inside the indicated ROIs. On average, the stretchable coil improved SNR within the central ROI from 135.2 to 558.3 (by 413%), within the right ROI from 144.8 to 685.5 (by 473%), within the left ROI from 135.5 to 564.1 (by 416%), within the anterior ROI from 137.9 to 597.5 (by 433%), and within the posterior ROI from 147.3 to 575.3 (by 390%).

### 3.3. In Vivo Imaging

**Figure 6:**
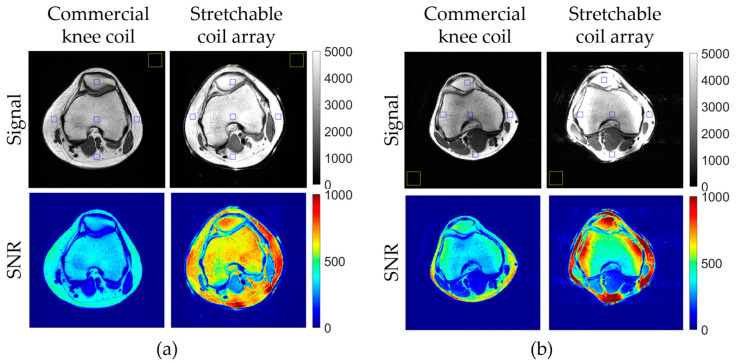
In vivo central axial knee images: signal (grayscale) and SNR (colormap), measured in two healthy volunteers. (**a**) Volunteer #1. (**b**) Volunteer #2. Yellow squares indicate the positions where background noise was evaluated.

In vivo imaging results, illustrated in Figure 6, demonstrate the signal and SNR superiority of the stretchable coil array compared to the eight-channel rigid knee coil array. Figure 6 shows central axial T_1w_ knee images from two volunteers. Figure 6a shows signal images for central axial and sagittal slices, indicating a substantial improvement in SNR within the entire axial slice with the stretchable array. Blue squares mark the regions of interest (ROIs) in the anterior, posterior, left, right, and center positions, while the yellow square indicates where background noise was evaluated. Specifically, the stretchable coil array improved SNR within the central ROI by 189% in volunteer #1 and by 155% in volunteer #2, within the right ROI by 146% in volunteer #1 and by 405% in volunteer #2, within the left ROI by 203% in volunteer #1 and by 128% in volunteer #2, within the anterior ROI by 151% in volunteer #1 and by 160% in volunteer #2, and within the posterior ROI by 209% in volunteer #1 and by 289% in volunteer #2. The difference in SNR enhancement between the two volunteers can possibly be explained by the different knee sizes of the volunteers as well as different coil positioning.

**Figure 7:**
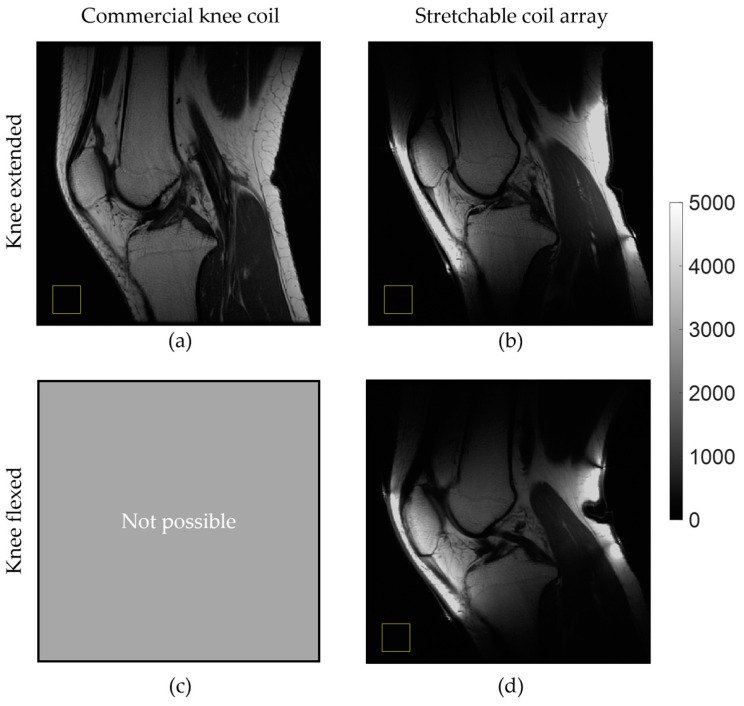
In vivo central sagittal knee images of a healthy volunteer using (**a**) a commercial rigid coil array and the stretchable coil array when the knee is (**b**) extended and (**d**) flexed. (**c**) Not possible to flex the knee inside the rigid coil array. Yellow squares indicate the positions where background noise was evaluated.

Figure 7 demonstrates the dynamic advantages of the stretchable array, comparing central sagittal T_1w_ knee images acquired in volunteer #1 using the commercial rigid and stretchable arrays. When comparing Figure 6a,b, the stretchable coil provides more signal due to its tighter fit while also allowing for image acquisition when the knee is flexed (approximately 30 degrees). The standard rigid coil array does not allow for this type of dynamic imaging (Figure 7c).

## 4. Discussion

This work demonstrates the design and feasibility of a self-tuning, multi-channel, stretchable coil array, in both simulation and imaging of a phantom and of human subjects. The negligible impact of the stretch on the resonance frequency, its consistent in vitro performance, and the substantial SNR improvements in vivo underscore the potential clinical relevance of this innovative coil design. The ability to maintain stable imaging performance across various stretching and loading conditions suggests applicability of the stretchable array in dynamic MRI applications.

In particular, the current design demonstrates exceptional stability in resonance frequency across various stretching and loading conditions with a frequency variation of less than 1%. The self-tuning interdigital capacitor geometry effectively counters the inherent frequency shift associated with stretching, ensuring consistent imaging performance. This feature is crucial for maintaining high SNR, especially in dynamic imaging scenarios. This work also demonstrated that the previously proposed self-tuning single-element coil design [39] retained its frequency stability when implemented in a multi-element linear array. Moreover, in vitro experiments validate the simulation results, showcasing consistent sensitivity (SNR) across individual coil elements and the combined coil array. In vivo imaging experiments demonstrate a substantial improvement in SNR when compared to a standard rigid knee coil array. This enhancement was observed both in the entire axial slice and within smaller ROIs. The ability to adapt to different knee positions further emphasizes the clinical potential of the stretchable concept.

Although the stretchable coil array showed improved SNR performance over the commercial coil array, the number of coil elements for the two arrays was not the same (six channels for the stretchable, versus eight channels for the commercial one), and thus the SNR improvement can be different with a higher channel count of the stretchable coil array. The slight sensitivity differences between the stretchable coil elements within the multi-channel array may be due to differences in their stretching states. Elements that are more stretched might experience different inductive and capacitive properties compared to less stretched elements, leading to non-identical sensitivity profiles among the coil elements. It may mean that coil sensitivities obtained at one knee position cannot be applied directly at other positions. In the future, we are planning to conduct extended in vivo trials involving a diverse range of healthy volunteers of different sex, age, BMI, and body types. This will ensure the coil array performs well across a broad spectrum of clinical scenarios. Moreover, extended in vivo trials in a range of patient categories can provide insights into the practical impact of differences in coil sensitivities and help refine the coil design.

Another limitation of the current stretchable array design is that stretching can only be performed in one direction (parallel to the direction of the array) for the self-tuning functionality to be used. This constraint limits the flexibility of the coil in adapting to complex anatomical contours that require multi-directional stretching. For instance, while the coil performs well when stretched along the primary axis, its performance degrades if significant stretching is required in orthogonal or oblique directions. In the future, we plan to address this limitation by investigating a matrix rather than a linear coil array, performing stretching tests in other directions, as well as investigating other smart coil geometries that can allow for stretching in two or even three directions [44].

In this study, we only demonstrated knee imaging at full extension and flexion of approximately 30° (see Figure 7). However, it is important to investigate how the stretchable coil performs in various flexion scenarios. To address this, we plan to build an MRI-compatible knee support for flexion testing, similar to the one in the study [33], that allows imaging in a set of fixed flexion angles or in a continuous kinematic way.

Although the described DIW fabrication technique provides fast and consistent results, it can be further improved. The number of fabrication steps can be reduced by printing the liquid metal directly onto the substrate [45], embedding the liquid metal within the substrate before it cures [46], or fabricating on other types of substrate materials such as stretchable fabrics [47]. When working with GaIn alloys, it is important to remember that, when exposed to oxygen in standard conditions, they immediately form an oxide layer which lowers the surface tension (e.g., from 625 mN/m to 356 mN/m for pristine eGaIn [48]), facilitating the formation of stable structures [49]. Due to such unique properties, GaIn liquid metal can be used in DIW when the flow rate, nozzle inner diameter, and standoff distance are optimized [50]. More economical Ga-based liquid metal alloys, e.g., GaSn and GaSnZn, can be used instead of GaIn, as indium is a relatively expensive and scarce material [51]. While considering alternative conductive materials for MRI RF coils, it is crucial to maximize the electrical conductivity of the material as it directly relates to the SNR of the acquired images [52]. Although the electrical conductivity of pure Ga (6.73 × 10^6^ S/m) is higher than any of its alloys [53,54], it needs to be mixed with other components to remain in liquid phase at room temperature. In recent years, so called “liquid–solid biphasic conductors” that combine Ga-based liquid-phase conductors and solid-phase particles and polymers are gaining momentum. These bi-phasic materials provide structural support, improve handling and deposition, increase electrical conductivity, and reduce surface tension, while remaining stretchable and mechanically robust [55].

Our experience shows that the coil elements maintain their shape and performance while being tested and stored under standard laboratory conditions. However, it is important to systematically analyze the durability and mechanical stability of the coil elements over extended periods and repeated use. Continuous stretching and relaxation cycles could lead to material fatigue, affecting the coil’s performance and lifespan. A systematic assessment of mechanical stability of the coil elements will be part of future studies.

Extensive clinical validation is essential to assess practical performance in diverse patient populations and imaging scenarios. This includes investigating its efficacy in different anatomical regions and patient conditions to establish broader applicability. Future research will explore real-time imaging scenarios, motion studies, imaging sequence optimization, and expansion into a 2D array.

## 5. Conclusions

In conclusion, our research presents a multi-channel, stretchable, self-tuning, liquid metal-based RF receive coil array for MRI, demonstrating promising outcomes in numerical simulations, in vitro experiments, and in vivo imaging. The coil’s stability in terms of resonance frequency (<1% variation) and sensitivity (<6% variation) during stretching was demonstrated in numerical simulations. Measured in vitro sensitivity, and substantial 1.5–4 times SNR improvements compared to a rigid commercial coil, underscore the potential clinical relevance of the stretchable coil array. Furthermore, dynamic imaging was investigated using the stretchable coil array to render imaging of a knee in flexion and extension, which is typically infeasible with rigid commercial coils. The broader impact of these findings on MRI technology and patient care is significant, as the stretchable coil array provides a more adaptable and form-fitting solution compared to traditional rigid coils. This technology has the potential to improve the diagnostic capabilities of MRI, especially in applications requiring movement or positioning changes during the scan. These advancements pave the way for future research and development in flexible RF receive coil arrays, ultimately contributing to improved diagnostic accuracy and patient outcomes in the field of medical imaging.

## Data Availability

The data presented in this study are available online via Figshare https://doi.org/10.6084/m9.figshare.26156164.v1. Dataset posted on 2 July 2024.

## Supplementary Materials

The following supporting information can be downloaded at: https://www.mdpi.com/article/10.3390/ma17133325/s1, Figure S1. 3D simulation model preparation steps. (a) Parameters to mathematically represent uniaxial coil stretching. (b) Coil element built based on these parameters when stretching is 30%. (c) Building a curved coil element using the wrap feature in SOLIDWORKS. (d) Curved coil element imported as an STL file in COMSOL. (e) Coil array surrounding a homogeneous phantom. (f) Full electromagnetic simulation model showing coil array, phantom, and surrounding air domain., Figure S2. Simulated individual sensitivity maps of each coil element. Figure S3. DIW fabrication process diagram. Figure S4. Fabricated coil element (a) before and (b) after liquid metal injection. The white scale bar is 1cm. Reference [56] is cited in the Supplementary Materials.
