# Supplementary material for: Design and Dynamic In Vivo Validation of a Multi-Channel Stretchable Liquid Metal Coil Array
Source: Materials (Basel). 2024 Jul 5;17(13):3325. doi: 10.3390/ma17133325 (PMC11243347; doi:10.3390/ma17133325)
Supplement: Supplementary file 1 [file materials-17-03325-s001.zip › materials-3081693-supplementary.pdf]

Supplementary Material

Design and dynamic in vivo validation of a multi-channel stretchable liquid metal coil array

Elizaveta Motovilova[1,2,*], Terry Ching[3,4,5], Jana Vincent[6], Ek Tsoon Tan[2], Victor Taracila[6], Fraser Robb[6], Michinao Hashimoto[3,4], Darryl B. Sneag[2], Simone Angela Winkler[1,*]

[1] Department of Radiology, Weill Cornell Medicine, New York, NY 10065, USA
[2] Department of Radiology and Imaging, Hospital for Special Surgery, New York, NY 10021, USA
[3] Pillar of Engineering Product Development, Singapore University of Technology and Design, Singapore 487372, Singapore
[4] Digital Manufacturing and Design (DManD) Centre, Singapore University of Technology and Design, Singapore 487372, Singapore
[5] Department of Biomedical Engineering, National University of Singapore, Singapore 117583, Singapore
[6] GE Healthcare, Aurora, Ohio, 44202, USA
* Correspondence: elm4010@med.cornell.edu (E.M.); ssw4001@med.cornell.edu (S.A.W.)

S1. 3D simulation model preparation

COMSOL software does not have a geometry wrapping feature and thus a two-step 3D model preparation process was used in combination with SOLIDWORKS: in the first step, the coil geometry was drawn in SOLIDWORKS and exported as an STL file; and in the second step, full-wave electromagnetic simulations of the coil were performed using COMSOL. Figure 1S shows the described process in more detail. First, all coil dimensions are parameterized to mathematically represent stretching in x-direction using a dimensionless parameter λ_x ranging from 1 to 1.3 and thus representing stretching from 0% to 30%. Assuming the materials are elastically homogenous and virtually incompressible, the following constraint is imposed $\lambda_x \cdot \lambda_y \cdot \lambda_z = 1$, from where it follows that *y* and *z* dimensions will change (shrink) according to $\lambda_y = \lambda_z = 1/\sqrt{\lambda_x}$. Thus, all changes in coil dimensions due to stretching in x-direction can be expressed in terms of the dimensionless parameter λ_x. Such parametrization is a mathematical approximation of a uniaxial coil elongation that allows to recreate coil geometry change due to stretching in a quick and efficient way. In this particular example shown below, the coil stretching equals to 30%. In the next step, a wrap function is used to bend the coil around a cylinder of the corresponding size. The resulting curved coil is then exported as an STL file and imported in COMSOL for further numerical electromagnetic simulations. In COMSOL, the single curved coil element is then copied and rotated to form a 6-channel coil array. The conducting traces were implemented as perfect electric conductors (PEC) of 0.5 mm×0.5 mm cross-section, embedded in a dielectric polymer substrate (dielectric constant, ε = 2.7) of 1 mm thickness. The coil array was positioned around a homogeneous cylindrical phantom (dielectric constant, ε = 78, electrical conductivity, σ=0.46 S/m). The coil array was simulated in various stretching configurations using cylindrical phantoms with a height of 150 mm and different diameters ranging from D_0 = 110 mm to D_6 = 143 mm, representing varied degrees of stretch from 0% to 30%, with a step size of 5%. The coil array and the cylindrical phantom are surrounded by a spherical Air Domain with Perfectly Matched Layers (PML) conditions and the domain boarders. Each coil element has one tuning (9 pF) and one matching (7 pF) capacitor and is terminated with a lumped port. Physics-controlled meshing is used with “Finer” element size option. Frequency Domain solver is used in the frequency range of 124 MHz to 132 MHz with a step size of 0.1 MHz.

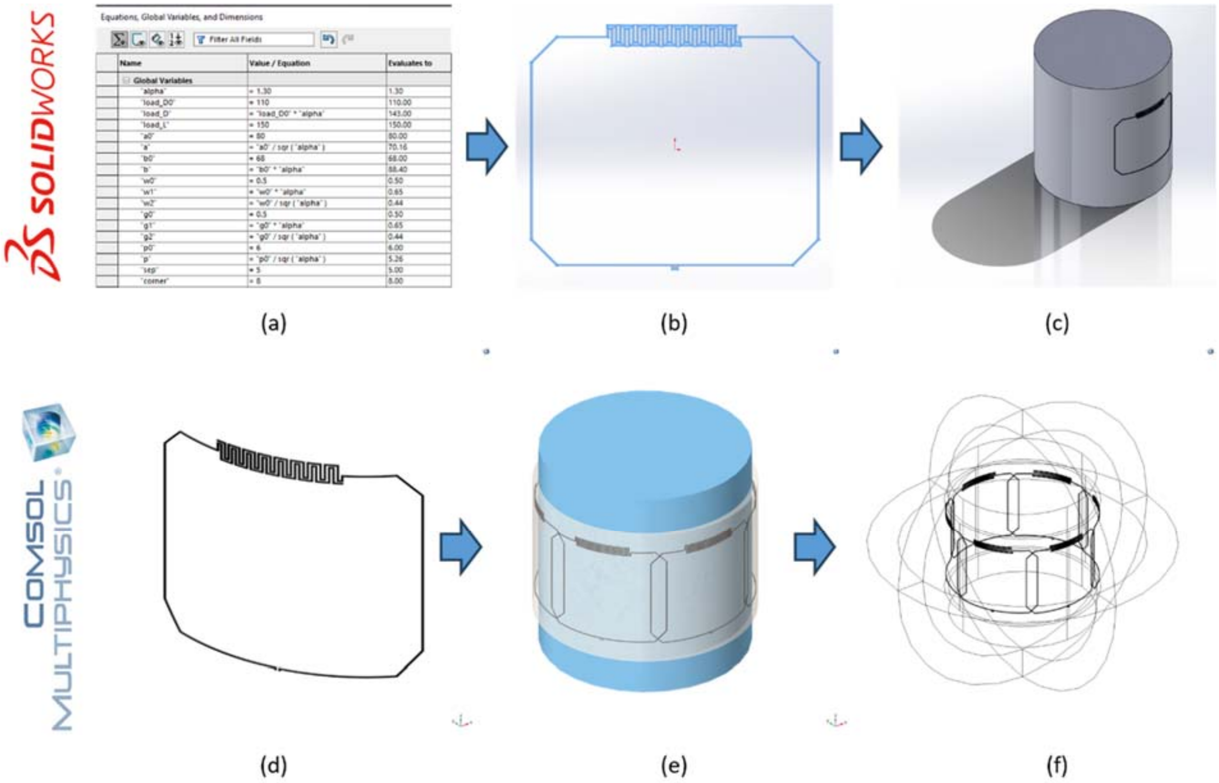

Figure S1. 3D simulation model preparation steps. (a) Parameters to mathematically represent uniaxial coil stretching. (b) Coil element built based on these parameters when stretching is 30%. (c) Building a curved coil element using the wrap feature in SOLIDWORKS. (d) Curved coil element imported as an STL file in COMSOL. (e) Coil array surrounding a homogeneous phantom. (f) Full electromagnetic simulation model showing coil array, phantom, and surrounding air domain.

S2. Simulated individual sensitivity profiles

Figure S2 shows the simulated individual sensitivity ($|B_1^-|$) maps of each coil element. Slight asymmetry of the sensitivity map with respect to the center of the coil is due to the wavelength effect that increases with the B_0 field strength. This is a well-known effect primarily observed at ultra-high field MRI and can be explained by the presence of large conduction currents induced within the conducting sample which create out-of-phase contributions to the $\boldsymbol{B_1}$ field [1].

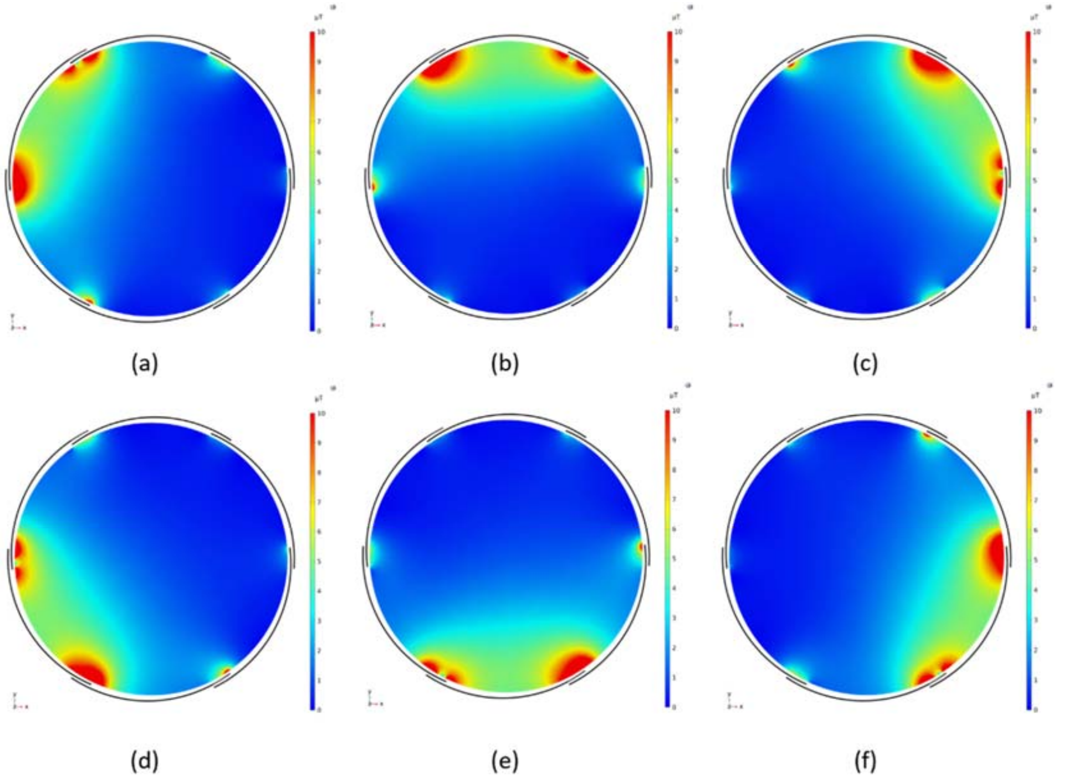

Figure S2. Simulated individual sensitivity maps of each coil element.

S3. Direct ink writing (DIW) fabrication process

Figure S3 shows a diagram of the DIW fabrication process. First, DragonSkin^{TM} 30 silicone (Smooth-On, Macungie, PA, USA) was prepared by mixing part A and part B as per the manufacturer instructions. The mixture was then spin-coated on a glass panel to create a thin 0.3 mm substrate and left to fully cure at room temperature for 4 hours. A fast-curing SpeedSeal silicone (Selleys) was used as an ink to 3D print (SHOTmini200ΩX 3D printer, Musashi, Japan) microfluidic channels on top of the prepared cured DragonSkin^{TM} silicone substrate. The printing resolution was limited by the size of the dispensing nozzle at 0.2 mm. Another layer of prepared cured DragonSkin^{TM} silicone was placed on top to seal the fabricated microfluidic channels. Finally, GaIn liquid metal (Ga 75.5% / In 24.5%, Sigma-Aldrich, Merck, USA) was manually injected into the channels through a syringe to form conducting traces of the coil.

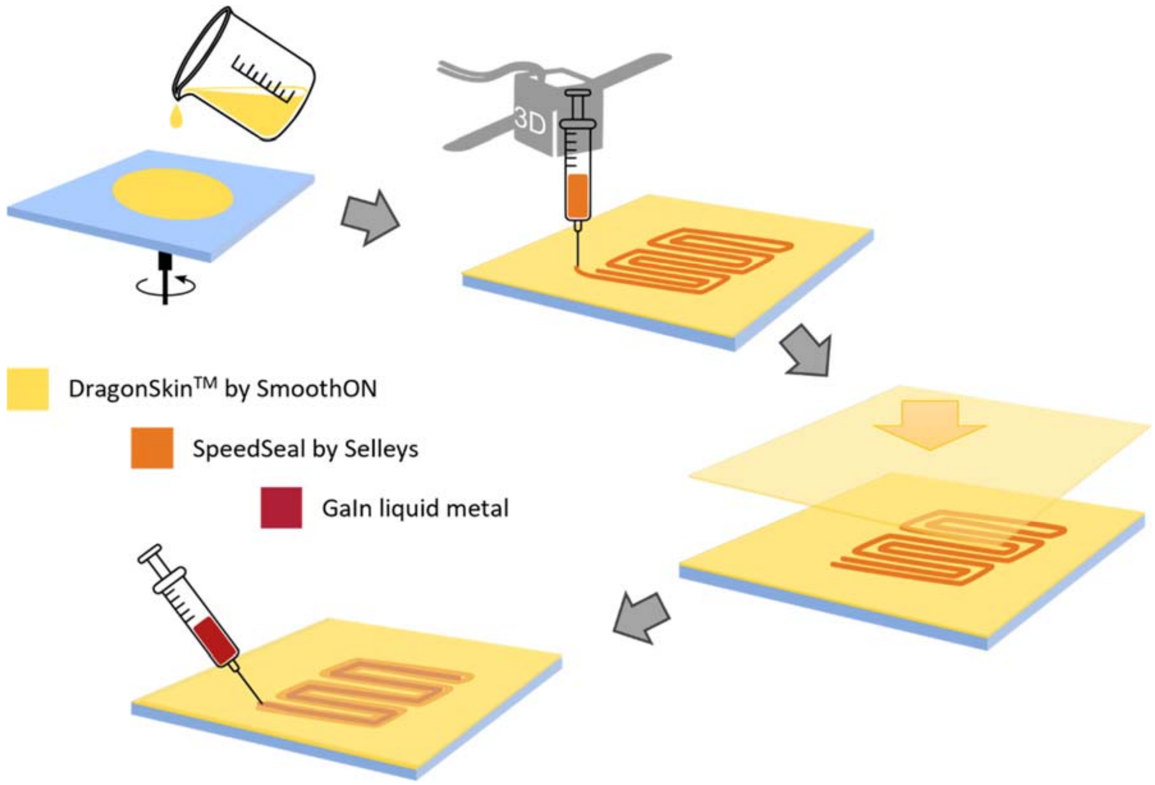

Figure S3. DIW fabrication process diagram.

S4. Fabricated coil element

Figure S4 shows photographs of the fabricated coil element taken against a black background when (a) only the silicone microfluidic channels are visible and (b) when they are filled with liquid metal and thin copper wires are inserted. At the insert point, the wires are parallel to each other with a separation of 1mm. The circuit board containing tuning, matching, and decoupling circuitry is then connected to the free ends of the wires. In our experience, the stretchability of the coil is not affected by the presence of the rigid circuit board as the stretching was only up to 30% and the wires were long enough to accommodate such small changes.

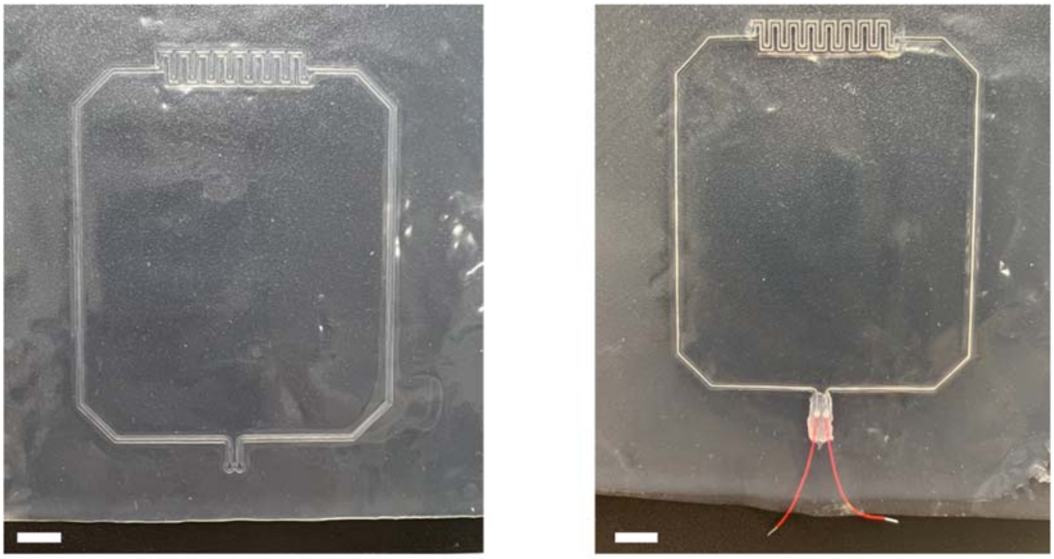

(a) (b)

Figure S4. Fabricated coil element (a) before and (b) after liquid metal injection.The white scale bar is 1cm.

References

1. Vaidya, M. V., C. M. Collins, D. K. Sodickson, R. Brown, G. C. Wiggins and R. Lattanzi. "Dependence of and field patterns of surface coils on the electrical properties of the sample and the mr operating frequency." *Concepts in Magnetic Resonance Part B: Magnetic Resonance Engineering* 46 (2016): 25-40.
